# Pathological Narcissism and Emotional Responses to Rejection: The Impact of Adult Attachment

**DOI:** 10.3389/fpsyg.2021.679168

**Published:** 2021-07-15

**Authors:** Samantha Reis, Elizabeth Huxley, Bryan Eng Yong Feng, Brin F. S. Grenyer

**Affiliations:** ^1^School of Psychology, University of Wollongong, Wollongong, NSW, Australia; ^2^Illawarra Health and Medical Research Institute, University of Wollongong, Wollongong, NSW, Australia

**Keywords:** narcissism, entitlement, adult attachment, rejection, emotional reactivity, affect

## Abstract

**Background:** Aspects of pathological narcissism, such as grandiosity, vulnerability and entitlement, tend be enacted in therapeutic settings, negatively influencing outcome and alliance between the clients and therapist. This research took an experimental approach to understanding the interplay between the emotional reactions of individuals with a pathological narcissistic presentation, and adult attachment style. We predicted that participants reporting narcissistic vulnerability would report greater insecurity in attachment (fearful and preoccupied styles), greater trait emotional reactivity, and also experience more intense and negative responses to simulated rejection

**Methods:** 269 participants (75.84% female, median age = 21) completed baseline and rejection trials of a virtual ball-tossing game, following the assessment of grandiose and vulnerable pathological narcissism, entitlement, adult attachment, trait emotional reactivity (measured prior to the rejection) and in-situ affective response (measured both before and after the rejection). Change in affect from baseline was calculated to capture affective responses to the manipulation.

**Results:** Vulnerable narcissism was positively associated with both fearful and preoccupied attachment, and negatively associated with secure and dismissive attachment, whilst grandiose narcissism was significantly related to preoccupied attachment only. Multiple hierarchical regression analyses showed vulnerable narcissism predicted both (1) more negative trait emotional reactivity and (2) a significant increase in negative affect following the rejection trial. Grandiose narcissism was associated with (1) higher positive trait emotional reactivity, and (2) significant reductions in positive affect following rejection.

**Conclusion:** Results indicated that those high in pathological narcissistic vulnerability reported greater insecurity in attachment, negative trait emotional reactivity and experienced a more negative and intense emotional reaction to rejection. Grandiose narcissism was related to a more deactivated pattern of emotional reactivity, and less positive (rather than more negative) emotional reactions. Findings have important implications for therapy, particularly regarding communication of emotions for individuals high in vulnerable and grandiose narcissism.

## Introduction

Narcissism is a personality construct associated with a range of interpersonal and emotional challenges (Kealy et al., 2017; Wright and Edershile, 2018), and can be manifested in pathological ways. Narcissism at low levels (‘normal’ narcissism) may function to protect and maintain self-image during challenging times and may contribute to self-esteem and wellbeing (Pincus et al., 2009). In extreme cases though, narcissism may involve more severe deficits in self and interpersonal functioning and may be expressed as Narcissistic Personality Disorder (NPD). Symptoms of NPD can include grandiosity, an inflated sense of self-importance, lack of empathy, manipulation/exploitation of others, entitlement, a sense of one’s own ‘specialness’, envy of others and perceptions that others are envious, a strong desire to be admired, and arrogant behavior including excessive bragging (American Psychiatric Association, 2013). Despite this seemingly impervious symptom profile, narcissism is thought to be driven by a need to protect a fragile sense of self (Gregg and Sedikides, 2010; Krizan and Johar, 2015), with these arrogant, conceited and self-aggrandizing behaviors used as a means to elicit approval from others, and mask deficits in self-worth. It has been noted, however, that NPD may be multidimensional in terms of its clinical presentation and that the current diagnostic criteria omits more ‘vulnerable’ narcissistic characteristics (Yakeley, 2018). Whilst grandiose pathological narcissism is characterized by factors traditionally associated with NPD, such as conceit, arrogance and a domineering attitudes towards others, vulnerable pathological narcissism is associated with feelings of anger, helplessness, shame and envy coupled with low self-esteem, interpersonal hypersensitivity, depression and in some cases, suicidality (Pincus et al., 2009, 2014; Tritt et al., 2010). Both subtypes have the same narcissistic function of drawing excessive attention from others, either due to the grandiosity or vulnerability displayed, and emanate from the same personality disorder. Vulnerable pathological narcissism tends to involve interpersonal problems associated with being too cold, avoidant and exploitable, in contrast to the grandiose presentation, which is characterized by problems with being too vindictive, domineering, intrusive and over-nurturant (Pincus et al., 2009). The clinical utility of differentiating these two dimensions has been well argued (Cain et al., 2008), with grandiosity linked to lower treatment utilization, poor therapeutic alliance, and therapist responses characterized by anger and hopelessness (Pincus et al., 2014; Ronningstam, 2017; Tanzilli and Gualco, 2020).

Despite research supporting the distinction between vulnerable and grandiose narcissism, until recently tools examining narcissism as both a personality trait (e.g., the Narcissistic Personality Inventory, Raskin and Hall, 1981) and clinically (the diagnostic criteria) predominantly assessed features associated with the grandiose narcissism, whilst omitting key themes associated with vulnerable narcissism (Tritt et al., 2010; Krizan and Johar, 2015; Wright and Edershile, 2018). In response, Pincus et al. (2009) developed the pathological narcissism inventory (PNI), a multidimensional tool to better capture both grandiose and vulnerable aspects of narcissism. The PNI includes subscales assessing different facets of pathological narcissism, with grandiosity comprised of scales capturing exploitation of others, self-sacrificing self-enhancement, grandiose fantasies and entitlement rage; and vulnerability reflected *via* contingent self-esteem, hiding the self and devaluing others (and a sense of shame associated with doing so). In addition to this, Krizan and Herlache (2018) have elaborated a narcissism spectrum model, an integrative framework which includes the concept of entitlement to explain how narcissistic traits are expressed and impact functioning. Core to this is the idea that narcissistic personality characteristics, particularly self-importance and entitlement, exist on spectrum between grandiosity and vulnerability. Entitlement is an important conceptual addition to formulations of narcissism, as it is considered to be the core of narcissism, expressed as either exhibitionism (grandiose presentation) or vulnerability depending on an individuals’ underlying temperament or personality (Miller et al., 2017; Wright and Edershile, 2018). Due to their impact on personality functioning both in early and later life, attachment experiences may comprise one of these underlying factors and influence the degree to which an individual experiences a vulnerable or grandiose presentation (Bennett, 2006; Meyer and Pilkonis, 2011) and narcissistic entitlement (e.g., Walters, 2019). Research has yet to examine the potentially dynamic relationship between constructs within the spectrum model (vulnerability, grandiosity and entitlement), adult attachment, and emotional reactivity. This study will investigate both trait emotional reactivity and in-situ affective reactions to a simulated experience of rejection within the context of this narcissistic spectrum model and adult attachment styles (Bartholomew and Horowitz, 1991).

### Pathological Narcissism and Emotion

Previous research on emotional responses to social exclusion and rejection has largely examined in this in relation to unidimensional rather than multidimensional constructions of narcissistic pathology, with some exceptions. Besser and Priel (2010) examined emotional reactions (anger and other negative emotional responses) to hypothetical scenarios of either achievement or interpersonal failure. Results indicated that that individuals higher in vulnerability were hypersensitive to scenarios involving imagined interpersonal rejection and displayed amplified expressions of anger and negative affectivity in response to these scenarios, whilst more grandiose presentations instead displayed amplified negative emotional reactions to imagined achievement setbacks. Distinctions between vulnerable and grandiose narcissism have also been demonstrated *via* examination of reactions to (imagined) private versus public negative events. Individuals high in vulnerability were found to show increased emotional distress from negative events played out in private settings, but those high in grandiosity demonstrated amplified negative emotional responses to experiences played out in public, with the association mediated by concerns about humiliation (Besser and Zeigler-Hill, 2010). This result seems consistent with the desire for impression management seen in grandiose profiles, and the need to maintain an illusion of superiority in front of others. Other studies have linked both pathological grandiose and vulnerable narcissism to self-reported aggression, but only the grandiose dimension was associated with aggressive behavioral responses and changes in testosterone level in response to ego threat (Lobbestael et al., 2014). These studies provide evidence of nuanced differences in emotional response, which vary according to context, for both grandiose and vulnerable pathological narcissism. However, changes in positive emotion were not captured within these studies, and measures used to assess pathological narcissism varied widely – with some studies focusing instead on ‘covert’ narcissism as an index of vulnerability (e.g., Besser and Priel, 2009; Brookes, 2015).

Other research has investigated ‘trait’ emotional reactivity for vulnerable, compared to grandiose narcissism (Sellbom et al., 2018). For example, Hyatt et al. (2018) found that both grandiosity and vulnerability were associated with anger reactions, but vulnerability this was more broadly related to negative affect, including sadness and shame. Taken together, individuals high in vulnerability are thought to experience greater negativity in their emotions more generally (e.g., Krizan and Johar, 2015; Zhang et al., 2017a), and also appear to experience amplified distress, dysphoria and sadness in response to threatening scenarios, particularly if the situation is interpersonal in nature (Besser and Priel, 2010; Besser and Zeigler-Hill, 2010; Hyatt et al., 2018).

Although negative emotional responses are commonly the focus of narcissism research, there is some evidence of positive trait emotionality for the grandiose profile. Grandiosity has been linked with high self-esteem, self-efficacy, extraversion and emotional regulation, with vulnerability showing the opposite relationship (Brookes, 2015; Rohmann et al., 2019; Kaufman et al., 2020). Whilst vulnerability has been associated with negative affective reactions and a propensity towards negative emotionality more generally negative (e.g., sadness, neuroticism) across multiple studies, the link between grandiosity and negative emotion is considerably weaker (Miller et al., 2011). Exploration of potential associations between pathological grandiose narcissism and both trait and state positive emotions may allow insight into the emotional nuances of individuals with this presentation.

### Pathological Narcissism and Adult Attachment Styles

Research has implicated insecure attachment broadly in the development and maintenance of personality pathology, including pathological narcissism presentations (Bennett, 2006; Maxwell and Huprich, 2014). Furthermore, attachment theory has been argued to be relevant to the treatment of pathological narcissism, and because of this, may help guide therapeutic approach (Bennett, 2006; Meyer and Pilkonis, 2011). Contemporary adult attachment frameworks (e.g., Bartholomew and Horowitz, 1991) theorize that distinct combinations of positive versus negative working models of self and other influence behavior, emotions and affective wellbeing in the interpersonal realm. Four attachment styles are thought to exist in adulthood: secure, insecure-preoccupied, insecure-fearful and insecure-dismissive attachment (Bartholomew and Horowitz, 1991). Secure attachment denotes a positive model of both the self and others, such that the individual has a strong sense of self-worth and trusts others to be caring and responsive in close relationships. In contrast, both the insecure-fearful avoidant and insecure-preoccupied attachment styles feature a negative model of self. In the case of preoccupied attachment, this is combined with a positive model of others, so others are highly valued and sought out as a source of validation to compensate for low self-worth. Similarly, individuals with a fearful attachment style experience a desire for relationships to compensate for low self-worth, but hold a negative working model of others, and so avoid close relationships for fear of rejection. The approach-avoidance conflict implicit in this attachment style can cause marked distress for the individual (Lopez, 1995; Reis and Grenyer, 2004b), and may contribute to the link between fearful attachment, psychopathology and treatment resistance in therapeutic settings (Reis and Grenyer, 2004b; Levy et al., 2011, 2018; Reiner et al., 2016; Petrowski et al., 2019). The insecure-dismissive avoidant style on the other hand, features a positive model of self, and a negative model of others. This combination of working models represents defensive independence – close relationships with others are not needed for maintenance self-worth, but at the same time, others are not trusted and relationships are avoided (Bartholomew and Horowitz, 1991; Griffin and Bartholomew, 1994).

Studies that have examined Bartholomew and Horowitz’s (1991) model of attachment in relation to pathological narcissism have yielded conflicting results and have utilized different models and measures of attachment. For example, some research has found no link between grandiosity and attachment (Kaufman et al., 2020), while other research links grandiosity to dismissive or ‘avoidant’ attachment more generally (Dickinson and Pincus, 2003; Zhang et al., 2017b). In contrast, vulnerable pathological narcissism appears to be more consistently associated with insecure attachment, and holding a negative working model of self. Studies using both clinical and nonclinical populations have linked narcissistic vulnerability to ‘anxious’ (mapping onto the preoccupied style) and ‘avoidant’ (mapping on to the fearful and/or dismissive style) attachment (Fossati et al., 2015; Kaufman et al., 2020). Others have more directly linked vulnerable narcissism to both preoccupied attachment (Dickinson and Pincus, 2003; Zhang et al., 2017b); and fearful attachment (Dickinson and Pincus, 2003). The idea that vulnerability may be characterized by either a more fearful or more preoccupied attachment style (or both) aligns with findings indicating that individuals high in narcissism are simultaneously anxious about maintaining attachments and reluctant to trust others (Fossati et al., 2015). This approach-avoidance conflict seems to suggest a more fearful attachment style. However, preoccupied attachment also seems congruent with a vulnerable presentation, as both concepts share a similar emphasis on gaining positive feedback from others in order to nurse a poor self-concept (Besser and Priel, 2009, 2010; Besser and Zeigler-Hill, 2010).

Measurement of attachment styles on one hand, and pathological narcissism on the other, has varied widely between studies, creating challenges when comparing results. However, findings of recent research utilizing the PNI (Pincus et al., 2009) and Bartholomew and Horowitz’s (1991) attachment model indicated that vulnerable pathological narcissism was positively related to both fearful and preoccupied attachment and negatively related to security in attachment, with this relationship mediated by feelings of shame (van Schie et al., 2021). In contrast, grandiose narcissism was positively related to secure attachment, but was unrelated to any of the three insecure attachment styles. These findings support the idea that individuals with a vulnerable pathological narcissistic presentation are more likely to experience attachment problems in adulthood, and that negative emotions (like shame) play a role in explaining the relationship between attachment and this vulnerability. The results of van Schie et al. (2021) also add to the somewhat conflicting findings regarding the relationship between grandiose pathological narcissism and adult attachment styles. It may be that attachment styles are comparatively more influential for individuals with a vulnerable narcissistic presentation than for those with a more grandiose one.

An exaggerated sense of entitlement, which may exist as part of both pathological grandiose and vulnerable presentations, may also be relevant to adult attachment style as it influences the quality and nature of interpersonal interactions. One study reported associations between psychological entitlement and a dismissive adult attachment style (Campbell et al., 2004). Results from Campbell et al. (2004) indicated that entitled behaviors, were acquisitive rather than defensive – they seemed to be motivated by greed rather than fear. Tolmacz and Mikulincer (2011) found that sense of entitlement was related to adult attachment insecurity in the form of attachment anxiety (indicating a preoccupied style) and avoidance (indicating a fearful and/or dismissive style). Aside from these findings, research on links between entitlement and adult attachment is limited, with much of the research on entitlement and adult attachment focusing instead on relational entitlement – the belief that one deserves more than their partner in romantic relationships – and childhood attachment to parents (e.g., Tolmacz, 2011; Tolmacz and Mikulincer, 2011; Rothman and Steil, 2012; Shadach et al., 2018). There are many reasons to expect that an exaggerated sense of entitlement may interact with adult attachment style and influence narcissistic pathology. For instance, a healthy sense of entitlement is thought to originate from caregiver interactions which are both sensitive and warm in emotional tone. These caregiver conditions are akin to those that are thought to generate attachment security which may translate to greater security in adulthood. Conversely, an exaggerated sense of entitlement (often seen in narcissistic presentations) has been theorized to emerge from parental behavior that is insensitive to needs, but occasionally over indulgent (Meyer, 1991; Tolmacz, 2011), and early trauma (Moses and Moses-Hrushovski, 1990). Whilst quality of early attachments to caregiver may be relevant to the development of entitlement, research addressing current attachment – particularly Bartholomew and Horowitz’s four category model – and entitlement as part of a narcissistic presentation is scarce.

### Pathological Narcissism, Attachment and Emotion

To date, few studies have integrated attachment in order to understand differences in emotional reactions across pathological narcissistic vulnerability and grandiosity. Besser and Priel (2009) are a notable exception, with findings from their two studies demonstrating associations between ‘covert’ narcissism, attachment anxiety (but not avoidance) and negative emotional states (anxiety, hostility and dysphoria) after an imagined romantic rejection. Although these results are promising, the lack of association between attachment avoidance and narcissism may be a function of collapsing the two avoidant attachment styles – fearful and dismissive – into one broad category of attachment ‘avoidance’ as measured by the Experiences in Close Relationships Revised scale (ECR-R; Fraley et al., 2000). Furthermore, whilst ‘covert’ narcissism may be similar to the vulnerable dimension of pathological narcissism it is not interchangeable, and tends to be measured using different tools – in this case the Hypersensitive Narcissism Scale (HSNS; Hendin and Cheek, 1997). Across studies of pathological narcissism there is little consensus on which measures best tap into pathological grandiosity and vulnerability, adding to conceptual confusion and precluding meaningful comparisons among studies.

This study aims to examine emotional reactions to experiences of rejection in relation to Bartholomew and Horowitz’s (1991) model of attachment and pathological narcissism. Self-reports of trait emotional reactivity will be utilized alongside Cyberball, a virtual ball-tossing game (Williams and Jarvis, 2006) to simulate an experience of rejection. Thus, both enduring tendencies to react in a particular way, and actual affective responses before and after a rejection experience will be used to provide a greater understanding of the emotional dynamics of pathological narcissism. Firstly, we hypothesized that narcissistic vulnerability would be positively associated with attachment styles featuring a negative model of self – preoccupied and fearful attachment. This prediction aligns with previous research and also the common focus on approval from others, sensitivity to rejection and low self-worth implicit in conceptualizations of narcissistic vulnerability, and both fearful and preoccupied adult attachment styles. Furthermore, it was predicted that individuals high in narcissistic vulnerability will have more negative trait emotional reactivity (measured *via* self-report) and also a more intense and negative response to simulated rejection (measured by change in affect from baseline). Based on previous research demonstrating that rejection and interpersonal relationships more generally are more salient to narcissistic vulnerability than grandiosity (Besser and Priel, 2009, 2010; Miller et al., 2011), and also that grandiosity may (under some conditions) be unrelated to attachment style (Kaufman et al., 2020), it was predicted that grandiosity would be unrelated to both trait emotional reactivity and affective changes in response to rejection. In this sense, individuals with a grandiose pathological narcissism presentation may have a more blunted emotional response to simulated rejection, as self-esteem may provide a buffer against these negative (and private) interpersonal experiences.

## Materials and Methods

### Participants

Participants were recruited from the community *via* social media and a university research site (*N* = 269). Participants engaged in university studies were provided course credit. The sample was comprised of *n* = 204 women (75.84%) and *n =* 65 men (24.16%) with a mean age of 23.68 years (*SD* = 8.12, Median = 21, range = 16–58). The sample primarily identified as Caucasian (68.40%), Asian (13.75%), and Hispanic (3.72%), with the remainder of the sample identifying as other (14.13%). Most participants were single (64.68%), with 21.93% stating they were in a relationship, 9.29% stating they were married and 4.09% divorced. Most participants (69.52%) were engaged in full time work or study, with the remainder of the sample engaged in part time work or study (23.05%) and not working or studying (7.44%). The study was approved by the University of Wollongong Human Research Ethics Committee (Protocol number 2017/171).

### Measures

#### Pathological Narcissism Inventory Super Brief

The 12-item pathological narcissism inventory super brief (PNI-SB) was utilized as a shortened measure of grandiose and vulnerable narcissism (Schoenleber et al., 2015). Participants responded on a 6-point Likert scale ranging from “Not At All Like Me” (0) to “Very Much Like Me” (5). The PNI-SB has demonstrated good criterion validity and internal consistency while retaining relevance to current theoretical understanding and literature (Schoenleber et al., 2015), and demonstrated adequate internal consistency for vulnerable (Cronbach’s *α* = 0.84) and grandiose (Cronbach’s *α* = 0.76) subscales in the current study.

#### Psychological Entitlement Scale

The psychological entitlement scale (PES) is a 9-item measure of entitlement and deservingness (Campbell et al., 2004). The items are measured on a 7-point Likert scale “strongly disagree” (1) to “strongly agree” (7). The PES demonstrated adequate internal consistency (Cronbach’s *α* = 0.85) and was included to provide a more comprehensive understanding of narcissism, in line with recent research which suggests that multiple measures may give a more comprehensive understanding of this complex and dimensional construct (Krizan and Herlache, 2018; Wright and Edershile, 2018).

#### Relationship Questionnaire

The 5-item relationship questionnaire (RQ) was used to measure adult attachment (Bartholomew and Horowitz, 1991). The four attachment styles are each represented by a single paragraph, which is rated “Not at all like me” (0) to “Very much like me” (100). A fifth categorical item ask participants to indicate which statement best describes them. The RQ is widely used and demonstrates content, construct, and discriminant validity across all four attachment styles (Bartholomew and Horowitz, 1991; Griffin and Bartholomew, 1994; Bäackström and Holmes, 2001).

#### Perth Emotional Reactivity Scale

The 30-item Perth emotional reactivity scale (PERS) used to assess trait emotional reactivity – how emotionally responsive participants tend to be generally, the intensity and duration of emotions, and the valence of emotional response (Becerra et al., 2019). Both positive (Cronbach’s *α* = 0.93) and negative (Cronbach’s *α* = 0.94) emotional reactivity were reliable in the current study.

#### Positive and Negative Affect Schedule-Expanded Form

A modified 20-item version of the Positive and Negative Affect Schedule-Expanded Form (PANAS-X; Watson and Clark, 1994) was used to measure positive and negative affective state. Sample items from the scale include “joyful” and “angry,” which respondents were required to rate on a 5-point Likert scale ranging from “Very Slightly or Not At All” (1) to “Extremely” (5). Items were grouped and summed according to valence of affect (e.g., sadness and anger as negative affect, joyful and happy as positive affect). This measure was administered following each Cyberball round.

#### Cyberball

Cyberball is an open-access program (retrieved from^1^) developed to simulate rejection/social exclusion. The Cyberball game screen in the present study involved one human player and 3 AI-controlled players which have been pre-programmed according to a baseline condition (completed first), and the rejection condition (completed second). The human player was named “You” while the AI-controlled players were each named “Player.” Cyberball has been effective in simulating inclusion and exclusion/rejection experiences, with the manipulation effects demonstrated across a number of studies (Williams and Jarvis, 2006; Renneberg et al., 2012; Gratz et al., 2013; Domsalla et al., 2014; Bungert et al., 2015; De Panfilis et al., 2015).

### Procedure

The study design is a within-subjects cross-sectional survey. Participants completed demographic questions and measures of narcissism, attachment and emotion reactivity, separated by baseline and rejection rounds of the Cyberball game (Williams and Jarvis, 2006).

Participants were advised that that they were playing the game with other people. On the first occasion they are passed the ball an equal number of times as other “players” in the game (baseline condition). Following completion of the PANAS-X to assess affective state, they are again asked to play Cyberball, but were this time subject to the rejection condition (where they are thrown the ball only once throughout the entire round). Each Cyberball condition was immediately followed by completion of the PANAS-X. PANAS-X scores were then used to calculate both the affective reaction to each condition (baseline and rejection) and also the change in affect from baseline. Participants were asked to answer a brief qualitative questions after each round as a manipulation check, and were excluded if they disclosed awareness of the manipulation.

## Results

### Descriptives, Correlations and Tests for Gender Differences

Descriptive statistics and correlations between variables are displayed in Table 1. Grandiose narcissism was negatively correlated with age, consistent with previous research indicating that increased age is associated with lower reported grandiose narcissism (e.g., Wilson and Sibley, 2011). As shown in Table 1, gender was not significantly correlated with narcissism, attachment styles, positive trait emotional reactivity or affective reactions to rejection, but was positively correlated with negative trait emotional reactivity. T-tests were conducted to further examine gender differences on the variables. The only significant gender difference was female participants (*M* = 3.40, *SD* = 0.90) who reported significantly higher negative trait emotional reactivity than male participants [*M* = 2.89, *SD* = 0.90, *t*(267) = −4.02, *p* < 0.001].

**Table 1:** Descriptive statistics for age, gender, attachment, narcissism, trait emotional reactivity, and affect after each Cyberball round.

S. No.	Variable	1	2	3	4	5	6	7	8	9	10	11	12	13	14	*M* (*SD*)
1.	Age															23.68 (8.12)
2.	Gender	0.01														1.76 (0.43)
Attachment																
3.	Secure	−0.11	−0.02													49.72 (29.47)
4.	Fearful	0.07	0.11	0.61^***^												54.32 (32.17)
5.	Preoccupied	−0.10	<0.01	−0.11	0.22^***^											50.39 (31.50)
6.	Dismissive	0.09	−0.11	0.13^*^	0.09	−0.26^***^										49.84 (29.19)
Narcissism																
7.	Grandiose	−0.18^**^	−0.06	0.04	0.06	0.33^***^	−0.04									3.98 (0.95)
8.	Vulnerable	−0.08	0.06	−0.30^***^	0.40^***^	0.49^***^	−0.21^***^	0.46^***^								3.32 (1.17)
9.	Entitlement	−0.04	−0.04	0.09	−0.06	0.02	0.22^***^	0.26^***^	0.02							2.70 (0.97)
Trait Emotional Reactivity (PERS)																
10.	Positive ER	−0.16^***^	0.08	0.44^***^	−0.35^***^	−0.09	<0.01	0.12	−0.29^***^	0.16^*^						3.55 (0.75)
11.	Negative ER	<0.01	0.24^***^	−0.29^***^	0.37^***^	0.34^***^	−0.11	0.24^***^	0.65^***^	0.02	−0.31^***^					3.28 (0.93)
Affective reactions (PANAS-X)																
12.	Positive baseline	−0.06	−0.10	0.31^***^	−0.24^***^	−0.14^*^	0.04	−0.04	−0.26^***^	0.07	0.43^***^	−0.33^***^				25.75 (8.17)
13.	Negative baseline	0.02	<0.01	−0.21^***^	0.32^***^	0.25^***^	−0.09	−0.06	0.37^***^	0.09	−0.25^***^	0.35^***^	−0.23^***^			14.40 (6.27)
14.	Positive rejection	−0.01	−0.09	0.29^***^	−0.23^***^	−0.13^*^	−0.08	−0.14^*^	−0.30^***^	0.07	0.36^***^	−0.33^***^	0.76^***^	−0.08		21.81 (8.29)
15.	Negative rejection	−0.09	−0.04	−0.09	0.26^***^	0.21^***^	−0.03	0.17^***^	0.41^***^	0.13^*^	−0.17^**^	0.40^***^	−0.07	0.74^***^	−0.11	15.94 (7.30)

*N* = 269. All regression coefficients are standardized.

* *p* < 0.05;

** *p* < 0.01;

*** *p* < 0.001.

Gender coded 1 = Male, 2 = Female. Positive baseline/negative baseline refers to positive and negative affect measured after Baseline round of Cyberball. Positive rejection/negative rejection refers to positive and negative affect after Rejection round of Cyberball.

Bolded values indicate significance at p < 0.05.

Results pertaining to self-reported trait emotional reactivity (measured by the PERS) and change in affect in response to rejection (measured by change in PANAS-X score from baseline) are reported separately below.

### Trait Emotional Reactivity

The relationship between narcissism, attachment and trait emotional reactivity (as measured by the PERS) was examined using multiple hierarchical regression analyses. Age, gender, and attachment styles were included in Step 1, followed by including narcissism variables in Step 2, to examine how narcissism is associated with emotional reactivity when controlling for variables in Step 1. The standardized regression coefficients are outlined in Table 2.

**Table 2:** Multiple hierarchical regression analyses predicting positive and negative emotional reactivity.

Step	Variable	Positive Emotional Reactivity β	Negative emotional reactivity β
1	Age	−0.12^*^	<0.01
	Gender	0.11^*^	0.21^***^
	Secure Attachment	0.36^***^	−0.13
	Fearful-avoidant Attachment	−0.14^a^	0.22^**^
	Preoccupied Attachment	−0.01	0.26^***^
	Dismissive-avoidant Attachment	0.07	−0.06
		*R*^2^ = 0.24	*R*^2^ = 0.26
2	Age	−0.11^*^	0.03
	Gender	0.13^*^	0.20^***^
	Secure Attachment	0.30^***^	−0.05
	Fearful-avoidant Attachment	−0.07	0.07
	Preoccupied Attachment	0.02	0.05
	Dismissive-avoidant Attachment	−0.01	0.02
	Grandiose Narcissism	0.21^**^	−0.05
	Vulnerable Narcissism	−0.30^***^	0.59^***^
	Psychological Entitlement	0.08	0.04
		Δ*R*^2^ = 0.07	Δ*R*^2^ = 0.21
		*R*^2^ = 0.31	*R*^2^ = 0.47

*N = 264.*

a *p* = 0.055;

* *p* < 0.05;

** *p* < 0.01;

*** *p* < 0.001.

Bolded values indicate significance at p < 0.05.

The full model predicting positive trait emotion reactivity explained 31% of the variance [*R*^2^ = 0.31, *F*(9, 255) = 12.77, *p <* 0.001], with the addition of narcissism Step 2 explaining 7% of the variance [Δ*R*^2^ = 0.07, Δ*F*(3, 255) = 8.35, *p <* 0.001]. Positive trait emotional reactivity was associated with gender (higher in women), secure attachment, higher grandiose narcissism, and lower vulnerable narcissism.

The full model predicting negative emotional reactivity explained 47% of the variance [*R*^2^ = 0.47, *F*(9, 255) = 25.46, *p <* 0.001], with the inclusion of narcissism explaining 21% of the variance [Δ*R*^2^ = 0.21, Δ*F*(3, 255) = 33.86, *p* < 0.001]. In Step 1, negative emotional reactivity was associated with higher fearful and preoccupied attachment. With the addition of narcissism in Step 2, the attachment styles became non-significant, and vulnerable narcissism was strongly associated with higher negative emotion reactivity. Gender was a significant predictor in both steps of the model with higher negative emotion reactivity associated with women.

### Change in Affect in Response to Rejection

#### Manipulation Check

The Cyberball manipulation was found to be effective in impacting affective experiences of participants. Paired t-tests were conducted to examine differences in positive and negative affect in the sample between the baseline and rejection round. Participants reported significantly less positive affect following the rejection (*M* = 21.81, *SD* = 8.29) compared to the baseline condition (*M* = 25.75, *SD* = 8.17), *t*(268) = −11.27, *p <* 0.001, and significantly higher negative affect following the rejection condition (*M* = 15.94, *SD* = 7.30), compared to the baseline condition (*M* = 14.40, *SD* = 6.27), *t*(268) = 5.08, *p <* 0.001.

To examine participant’s *change in affect* in response to rejection, reliable change indices (RCI) were calculated. RCIs are a commonly used technique allow for change between two points to be examined while accounting for measurement error (e.g., Stein et al., 2010; Zahra and Hedge, 2010). Regression analyses were then used to examine whether attachment styles and narcissism influence positive and negative affective reactions to the baseline and rejection conditions, and degree of affective change between conditions using dimensional RCI scores (Table 3). Using the cutoff of ± 1.96, 31.97% (*n* = 86) participants reported significant reliable change in either their positive or negative emotional state between conditions.

**Table 3:** Regression analyses predicting positive and negative affect after each condition and change in affect in response to rejection.

	Positive Emotions			Negative Emotions		
	Baseline	Rejection	RCI	Baseline	Rejection	RCI
Age	−0.05	−0.01	0.06	0.03	−0.07	−0.14^*^
Gender	−0.08	−0.09	−0.02	−0.06	−0.08	−0.05
Secure Attachment	0.25^***^	0.21^**^	−0.05	<0.01	0.14^*^	0.21^*^
Fearful-avoidant Attachment	0.01	0.02	0.01	0.21^**^	0.20^**^	0.03
Preoccupied Attachment	−0.04	<0.01	0.05	0.09	<0.01	−0.11
Dismissive-avoidant Attachment	0.01	−0.13^*^	−0.20^**^	−0.07	0.02	0.12
Grandiose Narcissism	0.03	−0.08	−0.16^*^	−0.16^*^	−0.09	0.07
Vulnerable Narcissism	−0.19^*^	−0.24^**^	0.06	0.31^***^	0.41^***^	0.22^**^
Psychological Entitlement	0.03	0.09	0.09	0.15^*^	0.13^*^	<0.01

*N* = 268. All regression coefficients are standardized.

* *p* < 0.05;

** *p* < 0.01;

*** *p* < 0.001.

RCI, Reliable Change Index.

Bolded values indicate significance at p < 0.05.

#### Positive Affect

The model predicting positive affect following baseline explained 14% of the variance [*R*^2^ = 0.14, *F*(9, 255) = 4.72, *p <* 0.001]. Positive emotions were associated with higher secure attachment style and lower vulnerable narcissism (Table 3). The model predicting positive emotions following rejection predicted 16% of the variance in positive emotion [*R*^2^ = 0.16, *F*(9, 255) = 5.44, *p <* 0.001]. Similar to the baseline condition, lower vulnerable narcissism and higher secure attachment were associated with positive emotion, however being male and dismissive attachment were also associated with lower positive emotion following rejection (Table 3). The model predicting change in positive emotion [*R*^2^ = 0.07, *F*(9, 255) = 1.97, *p* = 0.044] indicated that dismissive attachment and grandiose narcissism were significantly associated with greater reductions in positive emotions in the rejection condition compared to the baseline (Table 3). Overall, these findings suggest that although vulnerable narcissism is associated with lower positive emotion across baseline and rejection conditions, grandiose narcissism was associated with reduction in positive emotion between the conditions.

#### Negative Affect

The model predicting negative affect following baseline explained 21% of the variance [*R*^2^ = 0.21, *F*(9, 255) = 7.65, *p <* 0.001]. Higher negative affect following the baseline condition was significantly associated with greater fearful avoidant attachment, vulnerable narcissism and entitlement, and lower grandiose narcissism (Table 3). The model predicting negative affect following rejection predicted 22% of the variance [*R*^2^ = 0.22, *F*(9, 255) = 7.82, *p <* 0.001]. Following rejection, negative affect was significantly associated with greater secure and fearful-avoidant attachment, vulnerable narcissism and entitlement, and lower grandiose narcissism (Table 3). Finally, the model predicting change in negative affect [*R*^2^ = 0.10, *F*(9, 255) = 3.17, *p* = 0.001] indicated that significant increases in negative affect were associated with vulnerable narcissism and secure attachment (Table 3). Overall, these findings suggest vulnerable narcissism and entitlement were associated with greater negative emotion across the baseline and rejection conditions, however vulnerable narcissism was also associated with an increase in negative emotion between the conditions.

## Discussion

This study was the first to examine both trait emotional reactivity and responses to experimentally simulated rejection within pathological narcissism, whilst also taking into account Bartholomew and Horowitz’s (1991) four category model of adult attachment. In line with hypotheses, individuals with a more vulnerable narcissistic presentation evidenced a greater propensity towards fearful and preoccupied attachment – both attachment styles indicating a negative model of self. Conversely, vulnerable narcissism was negatively related to attachment styles denoting a *positive* model of self – dismissive and secure attachment. This result indicates that these attachment styles may be of particular relevance to the study of vulnerable pathological narcissism, and extends previous findings (e.g., Dickinson and Pincus, 2003; Smolewska and Dion, 2005; Fossati et al., 2015; Zhang et al., 2017b; van Schie et al., 2021). Furthermore, vulnerability was found to be associated with negative trait emotional reactivity, and results examining change in affect from baseline indicated that the experience of rejection was especially salient, negative and intense for those high in pathological narcissistic vulnerability. Although insecure attachment styles were found to be associated with negative trait emotional reactivity, these became non-significant when vulnerable narcissism was included in hierarchical regression models, highlighting the strength of the relationship between these constructs. These findings are consistent with previous research linking vulnerable narcissistic traits with emotional lability (Sellbom et al., 2018), and support the idea that the experience of rejection may be more difficult for individuals with a vulnerable pathological narcissistic presentation, and they may respond with greater emotional intensity when they feel slighted, rejected or criticized. Taken together, results suggest that a negative model of self (implicit in fearful and preoccupied attachment) is associated with more vulnerable narcissistic presentation which may play a role in generating more intense and affectively negative emotional reactions to interpersonal situations. Since causal models could not be tested within this study, the nature of the relationship remains a topic for future research.

In stark contrast, a grandiose narcissistic presentation was associated with positive trait emotional reactivity and also a reduction in positive emotions – rather than a change in negative emotions – in response to rejection. This finding could be interpreted as consistent with the suppression hypothesis around grandiosity, where affective responses are blunted to maintain a coherent and more positive/less worthless view of the self in the face of disconfirming information or experiences (Horvath and Morf, 2009). Alternatively, it may be that pathologically grandiose individuals *do* experience distress in response to social exclusion, but have difficulties reporting or recognizing distress. Research using the NPI (which predominantly measures grandiose narcissistic traits) alongside the Cyberball paradigm found that individuals who were high in narcissism displayed FMRI activity indicating distress in response to social exclusion (Cascio et al., 2015). Despite this, subjective self-reports showed no corresponding indications of distress. This finding indicates that whilst individuals who are high in (grandiose) narcissism may experience pain in response to rejection, this may not be captured in self-reports. This may be due to impression management needs, fears of appearing vulnerable in front of others, or an inability to subjectively recognize distress related emotions. The latter interpretation is supported by findings suggesting that individuals with NPD tend to have deficits in metacognitive ability, particularly around recognition of one’s own emotional states (Dimaggio et al., 2007).

Contrary to hypotheses predicting a lack of relationship between grandiose narcissism and attachment, grandiosity was positively associated with the preoccupied attachment style, which denotes a negative self and positive other combination of working models. Although a negative model of self is somewhat consistent with the conceptualization of grandiosity, the idealized view of others was unexpected (e.g., Dickinson and Pincus, 2003), especially given previous research indicating a lack of relationship between grandiosity and attachment. One explanation may be that, for individuals with pathological grandiose narcissism, others are idealized and sought out to provide reinforcement for arrogant and self-aggrandizing behaviors, which may require feedback to maintain. In this sense, those with a pathologically grandiose presentation may manifest a preoccupied adult attachment style, but more research is necessary to support this explanation. Alternatively, results may reflect conceptual cross-over between vulnerability and grandiosity, which were significantly correlated in this study, and previous literature (Krizan and Herlache, 2018).

Finally, psychological entitlement was associated with trait negative emotional reactivity, and a negative emotional response to *both* baseline and rejection rounds of the Cyberball game. Despite this, there was no significant relationship with change in affect from one round to the next, so negative emotions appeared to remain consistently low regardless of the rejection experience. Since previous research has shown that individuals with a strong sense of entitlement and grandiosity often display aggression in response to criticism (Campbell et al., 2004; Reidy et al., 2008), future research should seek to specify which types of negative emotions (e.g., sadness or anger based emotions) are experienced by individuals with an extreme sense of entitlement. Entitlement was also related to a dismissive adult attachment style, indicating a strong sense of self-worth combined with a negative view of others. This finding is consistent with findings of Campbell et al. (2004) and also with clinical data indicating that pathologically narcissistic individuals tend to manifest a dismissive attachment style (Diamond et al., 2014). The entitled view that one innately deserves more than others appears to be associated with pervasive but stable negative reactivity, and does not seem to be significantly changed by experiences of interpersonal rejection, according to present results.

The findings of the current study have important implications for narcissism and clinical practice. Vulnerable pathological narcissism mapped onto an adult attachment pattern featuring negative models of the self. Similar attachment patterns have emerged for individual experiencing depression (Reis and Grenyer, 2002, 2004a) and other psychopathology, like borderline personality disorder (Choi-Kain et al., 2009), emphasizing the risk of negative outcomes for individuals who are both high in narcissistic vulnerability and insecure in attachment style (Kaufman et al., 2020). The potential role of insecure attachment in the development and maintenance of pathological narcissistic vulnerability, and the influence this may have on emotional reactivity remains an important topic for longitudinal research.

Secondly, much research on narcissism has focused on negative emotions, however these findings suggest that positive emotions are also of importance. Rejection experiences were associated with a reduction in positive emotions, rather than an increase in negative emotions, for individuals reporting grandiose pathological narcissism. This highlights positive and negative emotions as correlated but conceptually distinct constructs (Serafini et al., 2016). A comprehensive understanding of this relationship may require further examination of positive emotion.

The findings of the current study also have implications for practice. Pathological narcissism often results in significant distress and impairment, and may inform treatment of NPD and other psychopathology, including depression and suicidality (Miller et al., 2007; Kealy et al., 2017; Kaufman et al., 2020). Based on these findings, individuals who are high in grandiosity may display more blunted or minimalistic emotional responses during therapy, particularly when recalling distressing interpersonal content. The idea that individuals with pathological narcissism have difficulty describing inner experience in therapy has been outlined elsewhere (e.g., Ronningstam, 2020; Dimaggio, in press), and may indicate an impaired ability to self-reflect. These blunted emotional responses may also extend to interpersonal events within therapy that represent a rejection type experience (e.g., termination of therapy). This may be a function of a more defensive emotional style, initially developed to project invulnerability and maintain the appearance of superiority in the eyes of others – in this case, the therapist (Kaufman et al., 2020).

The idea that grandiose individuals may have trouble expressing deeper emotional experience may also inform research around the negative effect grandiosity is thought to have on the therapeutic alliance (e.g., Zalman et al., 2019; Tanzilli and Gualco, 2020). Clients who are more grandiose in presentation may be less inclined to share their distress or divulge a need for help, placing strain on the alliance and leading to ruptures. Understanding these tendencies from an attachment perspective (i.e., as a deactivating/defensive behavior) may allow the therapist to more effectively work with the clients emotional responses, whilst modeling a secure attachment relationship.

Furthermore, clients with a pathologically grandiose presentation might benefit from strategies which target the ability to effectively communicate around negative emotional states, and promote genuine relatedness (Meyer and Pilkonis, 2011). The findings also highlight the importance of considering vulnerable narcissism in clients who may be more emotionally labile as part of their presentation. Other research has similarly highlighted emotional dysregulation and a weak sense of self as part of vulnerable narcissistic presentations (Kaufman et al., 2020; Huxley et al., 2021). Targeting these factors and prioritizing goals around the formation and maintenance of positive relationships may be beneficial. A nuanced approach to narcissistic presentations, informed by tendencies towards grandiosity and/or vulnerability, and awareness of their potential impact on alliance, may be helpful. Some recent research has elaborated on therapeutic strategies and approaches that are specific to clients presenting with pathological narcissism (e.g., Yakeley, 2018; King et al., 2020; Weinberg and Ronningstam, 2020; Dimaggio, in press) with promising results. This research suggests that tackling dysfunctional representations of self and others; impairments in ability to self-reflect; lack of agency; use of dysfunctional coping methods and defenses; problems with empathy and an impaired ability to mentalise, may provide a basis for treatment of individuals with pathological narcissism.

Some limitations should be considered when interpreting results. Firstly, the present research examined broad emotional reactions (positive or negative) using a subsection of self-report scales. Future research should aim to replicate using other measures of emotional reactivity, and potentially work towards identifying specific positive or negative emotions (e.g., disgust, anger, sadness) associated with responses to rejection for individuals high in grandiose or vulnerable narcissism. This avenue of research may be of particular relevance to pathological grandiose presentations, with some research indicating this is associated with marked aggression and accompanying physiological changes (Lobbestael et al., 2014). Since some research, including the current study, indicates more blunted (and sometimes even positive) emotionality for these individuals, while others indicates more aggressive responses to ego threat, future research should seek to untangle the conditions under which both positive and negative emotions may emerge. Findings of previous studies (e.g., Besser and Priel, 2010) suggest that grandiose individuals may be less attuned to interpersonal failures and more responsive to public and achievement based failures. Within the current study, participants were presented with an interpersonally themed rejection simulation which was experienced in private. This may provide some insight in to the lack of a strong emotional reaction to the task for individuals with a pathological grandiose presentation. Future research measuring both positive and negative physiological responses to different rejection scenarios could enhance understanding of emotional reactivity in this context. Examination of these relationships using a sample of individuals with NPD would also extend the clinical relevance of these findings.

The present research was also limited by the use of brief self-report measures of adult attachment relationship style and pathological narcissism. More lengthy interview based measures of attachment (e.g., the Adult Attachment Interview, George et al., 1985, unpublished) may provide greater insight into attachment processes, particularly those that stem from childhood experiences. Whilst adequate reliability was obtained for the PNI-SB, longer versions of the PNI may allow a more nuanced exploration of the sub-facets of vulnerable versus grandiose narcissism (e.g., hiding the self, contingent self-esteem) in relation to attachment and emotional reactivity.

Finally, there may be other factors at play that assist in understanding emotional reactivity of individuals with pathological narcissism, particularly in terms of experiences of rejection. For example, social rank motives may be activated when one feels inferior to others in a competitive context (e.g., in response to the Cyberball rejection). It has been shown that pathologically narcissistic individuals may be hypersensitive to social rank cues (Colle et al., 2020; Dimaggio, in press), and thus negative emotional responses to rejection may be a function of feelings of inferiority brought about by a perceived lower social rank than other ‘players’. Social rank motives may work alongside attachment processes to predict emotional reactions to rejection. Future research should seek to explore social rank in relation to emotional reactivity for individuals with a grandiose versus vulnerable pathological narcissistic presentation, with a particular focus on its relation to attachment.

This study represents the first experimental examination of emotional reactivity within pathological narcissism that has incorporated both Bartholomew and Horowitz’s (1991) adult attachment model, and entitlement. Vulnerable and grandiose forms of pathological narcissism showed distinct patterns of relationships with attachment and emotions, with vulnerability bearing a strong relationship to preoccupied and fearful attachment, negative trait emotional reactivity and increases in negative affect following rejection. In contrast, grandiose pathological narcissism was associated with preoccupied attachment, positive trait emotional reactivity, and a reduction in positive affect following rejection. Overall findings of this study are unique in that they not only identify attachment difficulties as broadly associated with narcissistic vulnerability, but also show that these factors may play a role in negative emotionality, and also negative emotional responses to experiences of rejection.

## Data Availability Statement

The datasets presented in this article are not readily available because participants within the study did not consent to sharing of data with individuals outside the research team. However, correlation tables can be made available by request. Requests to access the datasets should be directed to Samantha Reis, sreis@uow.edu.au.

## Ethics Statement

The studies involving human participants were reviewed and approved by University of Wollongong Human Research Ethics Committee. The patients/participants provided their written informed consent to participate in this study.

## Author Contributions

SR contributed to conceptualization and design of the study, wrote the first draft of the manuscript, and took charge of revisions. EH performed the statistical analysis, wrote sections of the manuscript, and contributed to revisions of the drafts. BE collected the data and was involved in the initial conceptualization of the project. BG contributed to manuscript revision and advised on the project. All authors contributed to manuscript revision, read, and approved the submitted version.

### Conflict of Interest

The authors declare that the research was conducted in the absence of any commercial or financial relationships that could be construed as a potential conflict of interest.

## Footnotes

^1^www.empirisoft.com/cyberball.aspx
